# The Brain Network of Expectancy and Uncertainty Processing

**DOI:** 10.1371/journal.pone.0040252

**Published:** 2012-07-02

**Authors:** Andrés Catena, José C. Perales, Alberto Megías, Antonio Cándido, Elvia Jara, Antonio Maldonado

**Affiliations:** Departamento de Psicología Experimental, Universidad de Granada, Granada, Spain; Bellvitge Biomedical Research Institute-IDIBELL, Spain

## Abstract

**Background:**

The Stimulus Preceding Negativity (SPN) is a non-motor slow cortical potential elicited by temporally predictable stimuli, customarily interpreted as a physiological index of expectancy. Its origin would be the brain activity responsible for generating the anticipatory mental representation of an expected upcoming event. The SPN manifests itself as a slow cortical potential with negative slope, growing in amplitude as the stimulus approximates. The *uncertainty hypothesis* we present here postulates that the SPN is linked to control-related areas in the prefrontal cortex that become more active before the occurrence of an upcoming outcome perceived as uncertain.

**Methods/Findings:**

We tested the uncertainty hypothesis by using a repeated measures design in a Human Contingency Learning task with two levels of uncertainty. In the high uncertainty condition, the outcome is unpredictable. In the mid uncertainty condition, the outcome can be learnt to be predicted in 75% of the trials. Our experiment shows that the Stimulus Preceding Negativity is larger for probabilistically unpredictable (uncertain) outcomes than for probabilistically predictable ones. sLoreta estimations of the brain activity preceding the outcome suggest that prefrontal and parietal areas can be involved in its generation. Prefrontal sites activation (Anterior Cingulate and Dorsolateral Prefrontal Cortex) seems to be related to the degree of uncertainty. Activation in posterior parietal areas, however, does not correlates with uncertainty.

**Conclusions/Significance:**

We suggest that the Stimulus Preceding Negativity reflects the attempt to predict the outcome, when posterior brain areas fail to generate a stable expectancy. Uncertainty is thus conceptualized, not just as the absence of learned expectancy, but as a state with psychological and physiological entity.

## Introduction

Expectancies are an essential part of human adaptation to the environment. The ability to anticipate future events allows us to organize our behavior in preparation for the impact of those events [1]. Nevertheless, expectancy is hardly ever perfect, so that most events occur in the world with some degree of uncertainty. In words of Baltasar Gracián (1637/1892), “[the wise man] may always hope for the best, but he always expects the worst, so as to receive what comes with equanimity” [2]. The present study is aimed at finding evidence about how, and where, uncertainty is computed in the brain.

Previous literature provides a plausible candidate to start our search. The stimulus preceding negativity (SPN) is a non-motor slow cortical potential (SCP) elicited by temporally predictable stimuli without any coupled motor requirements [3]–[4]. The SPN manifests itself as a slow cortical potential (SCP) with negative slope, growing in intensity as the stimulus approximates, and more clearly observed in parietal and precentral locations [5].

To date, the SPN has been hypothesized to be a physiological correlate of expectancy; that is, the origin of SPN would be the brain activity responsible for generating the anticipatory mental representation of an expected upcoming event. If this is true, the SPN must be the result of learning, so it will appear gradually, as the individual captures the regularities regarding the target stimulus in her environment. Most importantly, only those stimuli learnt to be predictable are expected to generate a significant SPN effect. Our proposal – henceforth, the *uncertainty hypothesis* – is just the opposite: it is not learned expectancy, but *uncertainty*, what mainly generates the SPN. We will try to show that such a hypothesis is compatible, not only with our own results, but also with the evidence available in previous literature (and, actually, it allows to reinterpreting previous results).

Expectancy is predominantly conceptualized as resulting from activation spread from the perceived predictive cue along an associative link, progressively generated during learning, provided that the cue and the outcome were contingently and contiguously presented [6]. Being unable to predict the upcoming event (the outcome) would preclude such facilitation/pre-activation, and thus uncertainty has been traditionally equated with the absence of any cognitive/physiological expectancy-related activity. Actually, a number of SPN results seem to support this prediction. For example, time estimation and decision-making tasks produce larger SPN amplitudes for informative than for non-informative outcomes [7]. SPN is also larger for rewards contingent on the people’s choice than for those administered gratuitously or at random [8]. Complementarily, in some studies, the effect has been observed to depend more directly on the predicted motivational value of the outcome than on its informative value [9], which has led some authors to advocate that the SPN results, at least partially, from the neural representation of the emotional value of the upcoming feedback, and not only from its perceptual representation [9]–[10].

In the case of a dichotomous event (present/absent), high predictability of either its presence or its absence implies low uncertainty. In the present experiment, only dichotomous events are used, so uncertainty equals probabilitistic non-predictability of the upcoming stimulus (correct/incorrect). In more complex tasks (see [9]) there can be more that two possible results of predictions, as, for example different categories representing degrees of prediction correctness. In these cases, uncertainty is defined as potential variability of the outcome. Other parameters being equal, the more possible results of a prediction there exist, the more uncertain the result will be.

However, it is important to make a distinction here between merely probabilistic and psychological uncertainty. For a probabilistically uncertain outcome to be also psychologically uncertain, a previous prediction must be at stake. In principle, we cannot say an individual is experiencing uncertainty about the future occurrence of an outcome if she was not trying to predict it. Both the generation of a prediction and the inability to confirm it in a consistent manner are necessary ingredients of psychological uncertainty. In addition, as it will be discussed later, although incentive and uncertainty are essentially different variables, the previous assertion also implies that uncertainty will produce neurocognitive effects in combination with the motivational relevance of the stimulus to be predicted.

Previous studies on SPN did not distinguish between probabilistic and psychological uncertainty. More specifically, control conditions, designed to avoid expectancy, probably generated disengagement from the task, and thus did not generate significant psychological uncertainty. In the experiment described here, we test whether the activity of the cortical sources supposedly involved in the SPN, depends on the degree to which an outcome is (un)expected.

Expectancy/uncertainty was generated by means of a one-cue one-outcome human contingency learning task (HCL, see [11]), in which the participant was asked to try to learn to predict the presence/absence of an outcome (a fictitious disease) on the basis of a cue (a fictitious medicine), on a trial-by-trial basis, and received a payoff for each prediction. We were mainly interested in the potential occurrence of the SPN in the interval between the learner’s prediction and the occurrence of the outcome (or its absence). Predictability of the outcome was controlled by manipulating the degree of contingency between the cue and the outcome. In accordance with the uncertainty hypothesis, we expect SPN to be larger when the outcome is unpredictable than when it is moderately predictable.

This HCL task presents several advantages over other tasks commonly used for studying ERP indices of expectancy. First, and most importantly, it allows a clear estimation of expectancy, and thus of uncertainty. Expectancy can be computed as the associative strength of associative models [12], or the conditional probabilities entering the probabilistic-contrast model [13], and uncertainty as an inverted U-shaped function over expectancy. In our case, low uncertainty corresponds to high expectancy and vice versa. And second, no contamination of motor or pre-motor activity is expected, as the target SPN interval begins once the response has already been made.

Parallel to the question of whether the SPN effect is linked to psychological uncertainty or not (and how to check it), is the issue of the neural source of such an effect. Converging evidence supports the existence of several cortical brain areas involved in the generation of SPN. First, dipole modeling [14]–[16] and fMRI studies [17]–[18] have identified the insular cortex as the main generator of SPN when feedback for a previous response conveys motivationally relevant information. Second, the anterior cingulate cortex (ACC) seems to be the best candidate generator when negative affect cues are used [15]. Third, some authors have proposed the involvement of dorsolateral prefrontal areas (DLPFC) in the anticipation of future events [5], [19]–[22]. And fourth, an increase in parietal cortex activation has been observed for temporal expectation tasks [23].

In contemporary learning theories, uncertainty is linked to sustained attention, and thus to the controlled effort to keep on learning when the events in the environment are not yet predictable. Therefore, we can tentatively hypothesize that the areas involved both in uncertainty computation and in SPN generation will be also related with cognitive control. Among the ones mentioned above, the best candidates are thus the ACC and the DLPFC. sLORETA will be used to try to cast some light on this issue. The potential role of other areas mentioned in the literature (insula, parietal cortex), in a broader network, will also be discussed.

In summary, the uncertainty hypothesis is related to the occurrence of the SPN in the interval between the prediction about an outcome and the occurrence of such an outcome. In contrast with the general assumption, we expect the SPN-Outcome amplitude to be larger in a condition in which outcomes are unpredictable (high uncertainty) than in one in which they are predictable (middle uncertainty). Complementarily, we postulate the prefrontal cortex as the best candidate of the source of that activation difference. The potential implication of other areas in the SPN, and its relation with previous results will be discussed.

## Materials and Methods

### Participants

Twenty-two Psychology students (2 left handed;14 women; median age: 20 years, range: 18–24) volunteered in the experiment in exchange for course credits. All had normal or corrected-to-normal vision, were healthy, were not currently medicated, and had not been previously diagnosed with any neurological disease. All participants signed an informed consent form approved by the Ethical Committee of the University of Granada and were treated in accordance with the Helsinki declaration.

### Apparatus, Stimuli and Procedure

**Figure 1:**
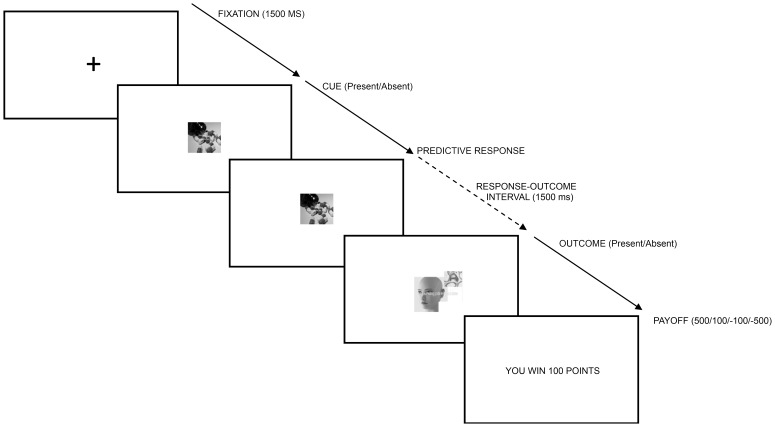
Timing of the events in each trial of the HCL learning task.

Participants seated in individual chambers, approximately 60 cm away from a 17in high-resolution LCD monitor, where all stimuli were presented. PC computers with Intel Core 2 Duo® processors were used for controlling the task and registering both behavioral and EEG data. The task was programmed, specifically for the present experiment, using Visual Basic 6®. The order of events in each task trial is depicted in Figure 1. Each trial presented the participant with a fictitious case of a person who had taken or not a certain drug (the cue), and later suffered or not a given side effect (the potential outcome). Between the cue and the outcome, the participant was asked to make a yes/no prediction about the occurrence of the outcome (“the side effect wil/will not occur”). The main manipulation involved the statistical relationship between the cue and the outcome, and thus the predictability of the outcome on the basis of the information provided on the presence or absence of the cue.

Each trial commenced with a central fixation point. After 1500 ms, information on the cue was provided, that is, the participant was informed whether the current case had taken the drug or not. The labels for the two cues in the two contingency conditions of the task were “perfluorato” and “dextroquinasa” (the assignation of labels to contingency conditions was balanced). The pictures indicating the presence or absence of the cue (a picture of several colored pills, or the same picture crossed off) were 300 pixels high and 400 pixels wide.

Once information on the cue had been presented, the participant predicted whether the outcome (the side effect) would occur or not. As soon as the prediction made, it was marked onscreen (yes/no), to prevent the participant to respond twice. Keys M and Z in the keyboard were assigned, in a balanced manner, to yes/no responses. All participants pressed the Z key with their left hand, and M key with their right hand. Although the cortical distribution of SCP could be changed by post-movement effects produced by the motor response preceding it, the balanced assignment of positive and negative responses to the right and left hands (which makes sure that, on average, participants respond the same number of times with the same hand on both conditions), ensures that any uncertainty effect on SCP will be unaffected by postmovement-related potentials. Once information on the cue had been presented, the participant predicted whether the outcome (the side effect) would occur or not. As soon as the prediction made, it was marked onscreen (yes/no), to prevent the participant to respond twice. Keys M and Z in the keyboard were assigned, in a balanced manner, to yes/no responses. All participants pressed the Z key with their left hand, and M key with their right hand. The cue remained onscreen until it was replaced by the outcome (1500 ms after the prediction). The participant was informed about the occurrence (or non-occurrence) of the side effect (the outcome) by means of a picture of a face showing or not the outcome and a written message (the labels for the two side effects were “pruritis” and “eritemia”, for the two contingency conditions, and the assignation of labels to contingency conditions was balanced). The picture and the message were replaced after 1500 ms by a payoff message. “You earn [lose] 100 [500] points”. All correct predictions were rewarded, and all incorrect ones were penalized; however, the amount of reward/penalty was selected randomly (100/500 points) in each trial. As noted above, this delayed payoff had no other function than extending the participant’s attention to the relevance of the feedback for a longer period. The intertrial interval varied randomly between 650 and 3000 ms.

The fixed 1500 ms prediction-outcome interval is justified on the basis of previous literature. On the one hand, most studies on the SPN set longer intervals (about 2000 ms), but differential effects are evident by the middle of that period (see [7]–[10]). On the other hand, long intervals make the detection of contingencies more difficult [11]. So, the interval was set to allow for contingency detection without affecting the possibility to detect differences between conditions in the SPN effect.

As noted above, the main manipulation involved the degree of statistical relationship (computed as *contingency*) between the drug and the side effect. In one condition, the side effect was predictable on the basis of the presence or absence of the drug; in the other, the occurrence of the side effect was not predictable from the presence or the absence of the drug. In other words, each participant did the task twice, with two different contingency levels (*ΔP* = .50; *ΔP*  = .00). Contingency (*ΔP*) is computed as the difference between the probability of the outcome in the presence of the cue [P(O/C)] and the probability of the outcome when the cue is absent [P(O/no C)]. In the positive contingency condition (hereafter, middle uncertainty, MidU, condition), the probability of occurrence of the outcome in patients who had taken the drug was.75, whereas the probability of the outcome in patients who had not taken the drug was.25. In the null contingency condition (hereafter, maximum uncertainty, MaxU, condition), on the other hand, both probabilities were.50.

Each contingency task consisted of 256 trials, and the task was interrupted after every 64 trial-block for the participant to judge the strength of the causal relationship between the cue and the outcome. Judgments were collected by using a scale bar with a cursor the participant could slide to indicate any value between -10 (the drug prevents the side effect to occur to a maximum degree) and +10 (the drug causes the side effect to a maximum degree), with the value 0 indicating that the drug neither caused nor prevented the side effect. In addition, the task was interrupted randomly (on average, after every 19 trials) by a screenshot, blank except for the sentence “Please let your eyes rest for 10 seconds”, after which the task resumed.

Both predictions and judgments were collected for analysis. Causal judgments were submitted to a 2 (Uncertainty condition: MaxU, MinU) x 4 (Block: 1–4) ANOVA. On the other hand, the observed probability of a positive prediction (“the side effect with occur”) in the presence of the drug, and the probability of positive prediction in the absence of the drug, were averaged for each 32-trial sub-block. These averaged observed probabilities were submitted to a 2 (Cue: cue-present trials, cue-absent trials) x 2 (Uncertainty condition: MaxU, MinU) x 8 (Sub-block: 1–8) within-subject ANOVA. In all cases, the Greenhouse-Geisser correction was used for assessing statistical significance.

### EEG Recording

EEGs were recorded from 61 scalp locations using tin electrodes arranged according to the extended 10–20 system mounted on an elastic cap (Brain Products, Inc), and referenced online to FCz. Vertical and horizontal eye activity were recorded from one monopolar electrode placed below the left eye, and two bipolar electrodes located in a straight line at the outer canthi of the left and right eye. Two of the scalp electrodes were attached to mastoids. All electrode impedances during recording were below 5 kΩ. EEG and EOG were sampled at 1000 Hz and amplified using a.016-1000 Hz band-pass filter. Subsequently, all EEG recordings were downsampled to 250 Hz, band-pass filtered using a.016–25 Hz 12db/octave, re-referenced offline to average activity of the mastoids electrodes, and FCz activity was recovered. Offline signal preprocessing was done using BrainVision Analyzer 2.0 software (Brain Products Inc, Munich, Germany).

### ERP Extraction and Analysis

Two 1900 ms segments were extracted offline. The first one (SPN-Outcome) was time-locked to the predictive response, with a 200 ms pre-response baseline. The segments lasted from 200 ms before the prediction to 200 ms post-outcome onset. The second one (SPN-Payoff) was time-locked to the onset of the outcome, with a 200 ms pre-outcome baseline, and so it lasted until 200 ms post-payoff onset. These SPN-Outcome and SPN-Payoff epochs were corrected for ocular artifacts using the Gratton-Coles algorithm [24]. Other artifacts were subsequently removed using an automatic rejection procedure: segments were excluded for the remaining analyses when amplitudes were outside the +/−100 µV range [25]. Please note that the filtering setting used here (.016–25 Hz) allows for significant drifting of the raw EEG, so that if a more restricted rejection criterion had been used, too many correct trials would have been unnecessarily rejected. On average, a 82% (minimum = 114) of trials were retained for further processing after the artifact correction procedure.

After artifact correction, an *SPN-Outcome score* was computed for each participant and condition, as the difference between the average amplitude in the interval between 1300 and 1500 ms after the predictive response (or, what amounts to be the same, during the 200 ms prior to outcome onset) and a baseline defined as the average amplitude between 1000 and 800 ms prior to outcome (see [7]–[8] for similar partitions). Similarly, an *SPN-Payoff* score was computed as the difference between the average amplitude between 1300 and 1500 ms after the outcome (the 200 ms prior to payoff onset), and a baseline defined as the average amplitude between 1000 and 800 ms prior to payoff.

Both SPN analyses were carried out on recordings from electrodes Fp1, Fpz, Fp2, F3, Fz, F4, FC3, FCz, FC4, C3, Cz, C4, P3, Pz and P4. SPN-Outcome scores were submitted to a 2 (Uncertainty: MidU and MaxU) x 5 (Location: Prefrontal, Frontal, Frontocentral, Central, and Parietal) x 3 (Laterality: Left, Middle, Right) multivariate analysis of variance (MANOVA; aimed to control for correlations between electrodes, [26]). SPN-Payoff scores were submitted to a 2 (Uncertainty: MaxU, MinU) x 2 (Valence: Positive and Negative payoff) x 5 (Location: Prefrontal, Frontal, Frontocentral, Central, and Parietal) x 3 (Laterality: Left, Middle, Right) multivariate analysis of variance. Statistical results are given as *F* approximations to Wilks’ *lambda*. Two-tailed paired samples t-tests were used for comparisons of interest. Task order was not included as a factor because neither main effects nor interactive ones were significant either in behavioral measures (prediction responses, all *p*>0.10; causal judgments, all *p*>0.16) or in SPN amplitudes (all *p*>0.41).

### Cortical Localization

Standardized Low-Resolution Electromagnetic Tomography (sLORETA, [27]–[28]) was used for estimating the 3-D cortical distribution of current density underlying scalp activity. In the current implementation of sLORETA, computations were done using the MNI152 template, with the 3-D space solution restricted to cortical gray matter, according to the probabilistic Talairach atlas. The cortical gray matter is partitioned in 6239 voxels at 5 mm spatial resolution. Brodmann anatomical labels are reported using MNI space. Standardized sLORETA current source densities with no regularization method were obtained from 60 channels for each participant in each condition and for each time point in the SPN-Outcome and SPN-Payoff time windows.

Source location followed a rationale adapted to the aim of the study: if current source density at a certain location is interpreted as an estimate of cortical activation, a significant correlation between current source density at a certain voxel and the magnitude of SPN (in a given uncertainty condition) can be taken as an indication of the involvement of such voxel in generation of the SPN (in that condition). Hence, the correlations between voxelwise current densities and SPN magnitudes can be used to identify the areas involved in the generation of SPN.

In summary, cortical localization analysis was carried out as follows. First, a single measure of the activation of each voxel (with a 5 mm spatial resolution) for the SPN interval was computed, by averaging voxel activations across that interval (200 ms preceding the outcome onset). Second, we computed the correlation between that estimated current density and the magnitude of the SPN effect, for each voxel and each condition, across participants. And third, those areas in which at least 10 voxels were found to significantly correlate with the SPN score were identified (see [29], for a similar procedure with behavioral data).

Finally, we were interested in checking how the estimated activations in the areas involved in SPN-outcome are related to one another, in order to get an idea of the possible shape of the brain network for expectancy and uncertainty processing (see [29], for a similar approach). Current source densities were first averaged across significant voxels within each previously identified brain area. The averages of the activations of the significant voxels within each selected brain area (see below) were submitted to a structural equation models analysis (SEM). SEM [30]–[31] serves purposes similar to multiple regression, but in a more powerful manner, as it allows to test whether the data are consistent with a model, including a causal one. A SEM model is composed by observed/latent variables and connection arrows. Directional arrows represent causal direction, and bidirectional arrows stands for correlation between the connected variables. SEM can be used both in a confirmatory way (to test the goodness-of-fit of an *a priori* defined model) and in an exploratory approach (i.e. to compare several models that usually differ in the number of connections). In both cases the goodness-of-fit indices test whether the proposed matrix of connections accounts for the observed pattern of variances/covariances. Thus, SEM is well suited to test the hypothesis that different brain networks account for the SPN differences observed as a function of our uncertainty manipulation. We made separate SEM analyses for each one of the two uncertainty conditions. As we do not have *a priori* information on the relationship between these areas, we tested 24 of all the possible models involving the brain locations selected after the cortical location step. Following literature recommendations [30]–[31], several goodness-of-fit tests were used to identify the most explanatory models: *Model Chi-square* (CMIN), *Root Mean Square Error of Approximation* (RMSEA), *Bentler-Bonett Normed Fit Index* (NFI), *Parsimony Normed Fit Index* (PNFI), and *Akaike Information Criteri*on (AIC). For a given set of models, we will consider one as better if it outperforms the others in at least three of these indices. Thus, for each uncertainty condition, we presented only the best fitting model.

## Results

### Behavioral Measures

**Figure 2:**
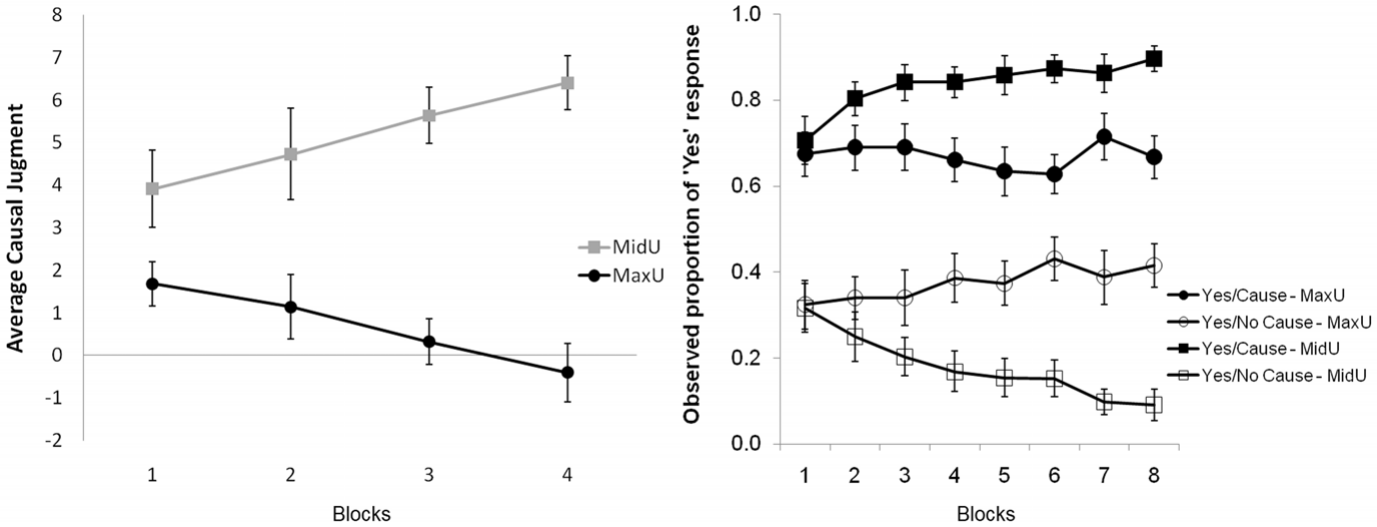
Behavioral results. Left panel: Mean causal judgments across the four 64-trial blocks, for the two uncertainty conditions (MaxU; MidU). Right panel: Probability of a positive prediction (“the outcome will occur”) in the presence and the absence of the cue, averaged across trials, for each 32-trial sub-block and the two uncertainty conditions. Error bars denote standard errors of the mean.

Two separate analyses of variance were done for causal judgments and predictions. The Greenhouse-Geisser correction was used for assessing statistical significance. A 2 (within subjects, Uncertainty: MidU, MaxU) x 4 (within subjects, Block:1–4) ANOVA on causal judgments yielded main effects of Uncertainty, *F*(1, 21) = 60.82, *MSE* = 14.58, *p*<.01, *η* ^2^ = .74, and the Uncertainty x Block interaction, *F*(3, 63) = 5.24, *MSE* = 8.42, *p*<.01, *η* ^2^ = .20. The left panel of Figure 2 displays mean judgments across blocks for the two Uncertainty conditions. Bonferroni post-hoc comparisons in the interaction yielded significant differences between the two Uncertainty conditions for blocks 2, 3, and 4, but not for Block 1 (*p* = .039). Trend analyses only revealed a linear component in the MidU condition, *F*(1, 21) = 6.90, *MSE* = 11.28, *p* = .02.

For the analysis of participants’ predictions, we computed the observed probability of a positive prediction (“the outcome will occur”) both in cue-present, *P*(Yes/Cue) and cue-absent, *P*(Yes/No cue) trials across 32-trial sub-blocks (8 sub-blocks per contingency condition). A 2 (within subjects, Uncertainty: MidU, MaxU) x 2 (within subjects, Cue: present, absent) x 8 (within subjects, Block: 1–8) ANOVA on those probabilities showed neat sensitivity to Uncertainty. On the one hand, positive predictions were more likely in cue-present than in cue-absent trials, *F*(1, 21) = 56.20, *MSE* = .69, *p*<.001, *η* ^2^ = .73, although that difference was larger, for MidU than for the MaxU condition, *F*(1, 21) = 5.39, *MSE* = .19, *p*<.01, *η* ^2^ = .57. As can be seen in the right panel of Figure 2, the probability of a positive prediction approached 1.00 in cause-present trials, and.00 in cause-absent trials in the MidU condition, whereas those probabilities remained far from the probability scale ends in the MaxU condition. In addition, this pattern becomes neater as training proceeds, as shown by a significant Uncertainty x Cue occurrence x Block interaction, *F*(7, 147) = 3.93, *MSE* = .04, *p*<.01, *η* ^2^ = .16. These results qualitatively match previous reports of experiments with a similar procedure (see [32]).

### SPN-Outcome

**Figure 3:**
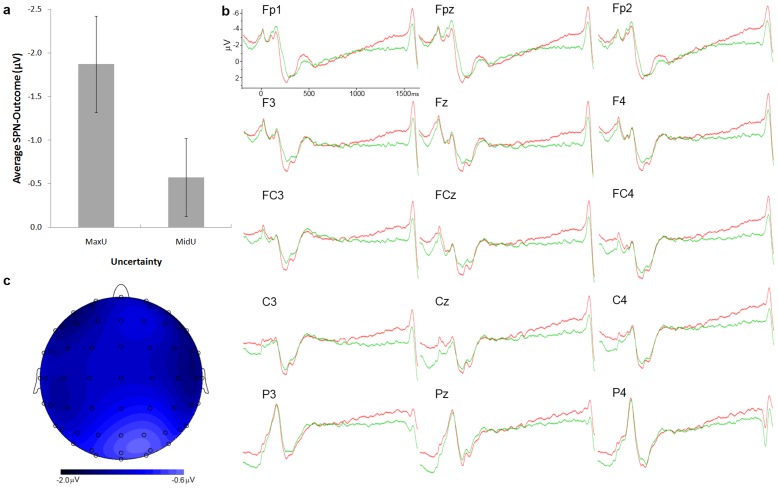
SPN-Outcome magnitude and SCP. (a) Magnitude of the SPN-Outcome for the two uncertainty conditions during the prediction-outcome interval. Error bars denote standard errors of the mean. (b) SCP waveforms for the prediction-outcome interval in the selected electrodes and the two uncertainty conditions (MaxU, MinU). (c) Topographical map of the MaxU-MidU difference.

SPN-Outcome scores were submitted to a 2 (Uncertainty: MidU and MaxU) x 5 (Location: Prefrontal, Frontal, Frontocentral, Central, and Parietal) x 3 (Laterality: Left, Middle, Right) multivariate analysis of variance. This analysis yielded significant main effects of Uncertainty, *F*(1,21) = 7.43, *p<*.02, *η^2^* = .26, Location, *F*(4,18) = 6.05, *p*<.01, *η^ 2^* = .57, and Laterality, *F*(2,20) = 11.68, *p<.01*, *η^ 2^* = .54. [The analysis of the SPN-Outcome after removing the two left-handed participants showed the same pattern of significance. Uncertainty: *F*(1,19) = 6.56, *p*<0.02; Location *F*(4,16) = 6.14, *p*<0.01, Laterality *F*(2,18) = 10.84, *p*<0.01. That was also the case for results regarding SPN-Payoff scores. Uncertainty x Value: *F*(1,19) = 8.76, *p*<0.01. Importantly, given that the ratio of left-hand and right-hand responses was the same for the two uncertainty conditions (left-hand: 133 and 133, right-hand: 134 and 126, respectively for MaxU and MidU conditions), it seems clear that post-movement potentials cannot account for SPN uncertainty differences.] 81.8% of participants showed less negative SPN scores (smaller in absolute value) for the MidU condition than for the MaxU one [*t*(21) = 2.73, *p*<.02]. According to Bonferroni correction, SPN scores for Frontopolar channels were higher than those for frontal sites (*p* = .02). SPN scores were lower for left than for central and right channels (all *p*<.01). No other differences were close to significance. Mean SPN-Outcome scores for the two conditions, averaged across sides and locations are displayed in Figure 3 (*a*). Figure 3 (*b*) displays the waveforms for the two uncertainty conditions (clearly showing a steeper and more pronounced SPN for the MaxU condition), for the selected electrodes, and the topographical map of the differences between MaxU and MidU conditions in the SPN interval (c).

As noted in the Methods section, a single measure of the activation of each voxel (with a 5 mm spatial resolution) for the SPN interval (200 ms preceding the outcome onset) was firstly computed, by averaging activations for that interval. Second, we computed the correlation between that estimated current density and the magnitude of the SPN effect, for each voxel and each condition, across participants. And third, those areas in which at least 10 voxels were found to correlate significantly with the SPN scores were identified.

Table 1 displays the list of areas identified by using this method. Brodmann areas and coordinates (MNI space) correspond to the voxels where current density-SPN relationship (*R* ^2^) was maximal, for each area and each condition. For the MaxU condition, current source density covaried with SPN magnitude in the DLPFC (BA9) the ACC (BA24), the insula (BA13), and the parietal cortex (BA40). In the MinU condition, current source density covaried with SPN magnitude in the Insula (BA13), and the parietal cortex (BA40).

**Table 1:** Brain locations in which current source density was significantly correlated (*R* ^2^) with SPN-Outcome score, at least in one of the two uncertainty conditions (MaxU, MinU).

Label	BA	k	X	Y	Z	R^2^
Max Uncertainty						
Inferior Frontal Gyrus	9	8	−50	0	25	0.30*
Insula	13	19	−30	−40	20	0.28*
Anterior Cingulate	24	26	−5	30	−5	0.27*
Supramarginal Gyrus	40	30	−55	−60	30	0.40*
Mid Uncertainty						
Inferior Frontal Gyrus	9	1	−45	10	30	0.10
Insula	13	15	−40	−40	20	0.29*
Cingulate Gyrus	24	1	−5	−20	40	0.14
Inferior Parietal	40	93	−45	−45	55	0.40*

Note: *p<.05. BA: Brodmann area. X, Y, and Z coordinates are in MNI space for the voxel with the maximal relationship with SPN-Outcome score (*R* ^2^). k is the cluster size in voxels.

### SPN-Payoff

**Figure 4:**
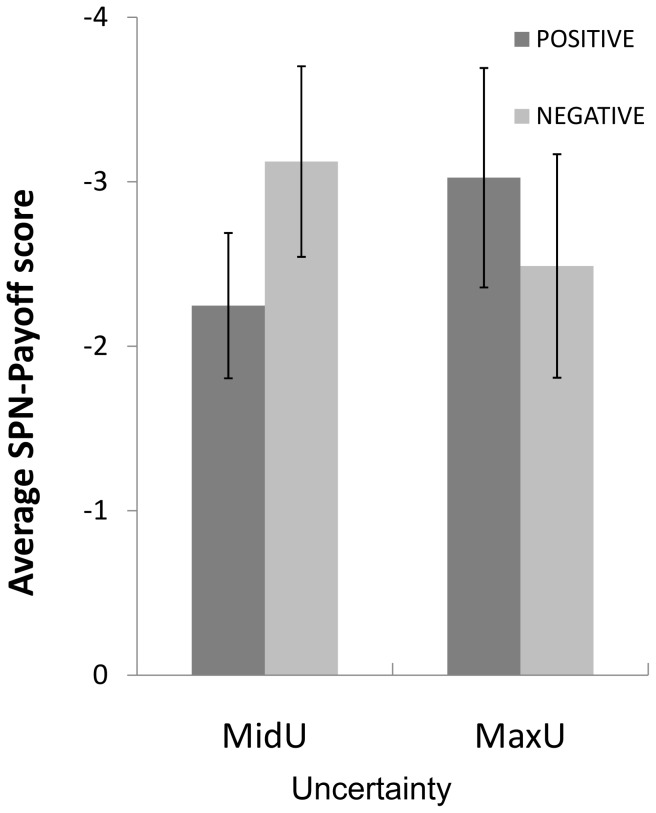
SPN-Payoff magnitude. Uncertainty x Payoff interaction on the SPN-Payoff score. Error bars denote standard errors of the mean.

SPN-Payoff scores were submitted to a 2 (Uncertainty: MaxU, MinU) x 2 (Valence: Positive and Negative payoff) x 5 (Location: Prefrontal, Frontal, Frontocentral, Central, and Parietal) x 3 (Laterality: Left, Middle, Right) multivariate analysis of variance. The MANOVA on the SPN-Payoff scores showed main effects of Location, *F*(4,18) = 13.38, *η^2^* = .75, *p*<.01, and Laterality, *F*(2, 20) = 16.87, *p*<.01, *η^2^* = .63. Bonferroni post-hoc tests showed that SPN-Payoff was larger at frontocentral, central and parietal channels than at the remaining locations (all *p*<.03), and at right and central than at left hemisphere channels (all *p*<.02). Neither feedback valence nor the uncertainty manipulation showed significant effects. However, there was a significant Uncertainty x Valence interaction, *F*(1,21) = 11.28, *p*<.01, *η^2^* = .35. The difference between positive and negative payoffs was not significant either for the MidU condition, *t*(21) = 1.86, *p* = .08 or for the MaxU one, *t*(21) = 1.63, *p* = .12. The SPN-Payoff score was significantly larger for MaxU than for MidU only for the positive Payoff condition, *t*(21) = 2.28, *p<* = .04 [see Figure 4].

As we did with the SPN-Outcome, we identified the areas in which the SPN-Payoff score (computed from positive feedback trials only, as the difference between MaxU and MinU conditions was significant only for these trials) covaried with current source density. As shown in Table 2, in the MidU condition, the SPN-Payoff score significantly correlated with estimated current densities in BA24, BA13 and BA40. On the other hand, in the MaxU condition, the SPN-Payoff significantly correlated with the estimated current density in BA39 and BA40.

**Table 2:** Brain locations in which current source density was significantly correlated (R^2^) with SPN-Payoff score, at least in one of the two uncertainty conditions (MaxU, MinU).

Anatomic Label	BA	k	X	Y	Z	R^2^
Max Uncertainty						
Inferior Parietal	40	104	−40	−65	45	.45*
	39	58	35	−65	40	.28*
Insula	13	1	45	−40	20	.05
Cingulate Gyrus	24	1	5	5	30	.15
Mid Uncertainty						
Inferior Parietal	40	45	−55	−30	25	.47*
	39	1	55	−60	25	.04
Insula	13	5	35	20	15	.25*
Cingulate Gyrus	24	67	20	−95	−15	.40*

Note: *p<.05. BA: Brodmann area. X, Y, and Z coordinates are in MNI space for the voxel with the maximal relationship with SPN-Payoff score (*R* ^2^). k is the cluster size in voxels.

### Structural Equations Modeling (SEM)

**Figure 5:**
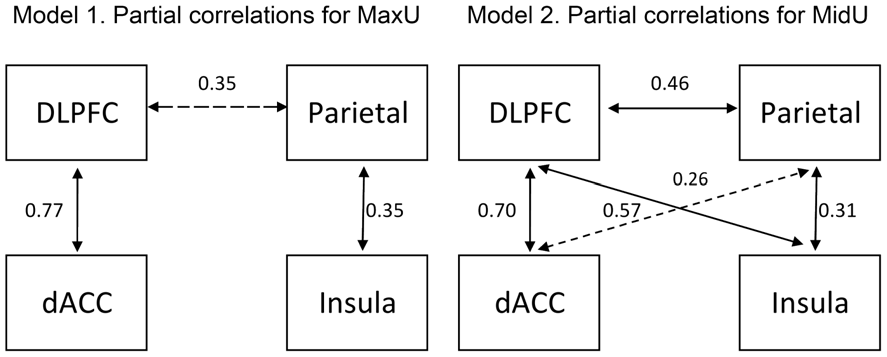
Graphical depiction of SEM results: brain networks for uncertainty and expectancy. Best-fitting models accounting for co-activation in the MaxU (top panel) and the MinU (bottom panel) conditions, according to the structural equation model (SEM) analysis. All the solid arrows are significant at *p*<.03. Dashed arrows, Model 1 *p* = .08, Model 2 *p* = .18.

For the SEM analysis, we used the average activity of significant voxels of the four areas involved in the generation of SPN-Outcome in the MaxU condition: dorsal ACC (BA24), DLPFC (BA9), Insula (BA13), and Parietal cortex (BA40). Following the rationale described in the Methods section, the model that best accounts for co-activation in the MaxU condition (Model 1) resulted to be the one depicted in the left panel of Figure 5. The one that best accounts for co-activation in the MidU condition is displayed in the right panel of Figure 5 (Model 2). Fitting parameters for the two models and the two conditions are shown in Table 3.

**Table 3:** Fitting-quality parameters, for the models of co-activation identified by SEM analysis^1^, and the two uncertainty conditions.

Model	NPAR	CMIN	RMSEA	NFI	PNFI	AIC
Maximum Uncertainty (MaxU)						
MaxU	7	2.34	.00	.94	.47	16.33
MidU	9	0.70	.00	.99	.16	18.69

1 NPAR, number of parameters in the model,;AIC, Akaike Information Criterion; CMIN, Chi-square; NFI, (Bentler-Bonett) normed fit index; PNFI, parsimony normed fit index; RMSEA, root mean square error of approximation.

## Discussion

### Summary of Results and Theoretical Implications

Behavioral results showed the expected pattern, which is a requisite for SCP interpretation. Both judgments and predictive responses showed that people discriminate between contingencies. In most other causal learning tasks (see [33] for a review), judgments in zero-contingency condition (MaxU) are slightly higher than in the present case, that is, people tend to perceive low to moderate positive contingencies where there is not any. Still, in our case, even judging the cue-outcome causal relation as virtually inexistent, participants kept on responding “yes” slightly more often in cue-present than in cue-absent trials (which can be a manifestation of the well-known bias in favor of positive contingency [34]).

Participants’ judgments and predictions fully corroborated their behavioral sensitivity to our key uncertainty manipulation. In parallel with this, SCP results show that the SPN-Outcome is larger when the upcoming event (the outcome) is uncertain than when it is relatively predictable. In the uncertain condition (MaxU, null contingency), its magnitude directly correlates with current source density in prefrontal areas (ACC and DLPFC). In the predictable condition (MidU,.50-contingency), however, the magnitude of the SPN correlates with estimated activity in the parietal cortex (BA40) and the insula (BA13). Complementarily, once the uncertainty is resolved, and the individual stays waiting for the corresponding payoff for her right or wrong prediction, the main effect of the contingency manipulation on the SPN score disappears, and only a significant feedback valence effect in the MidU condition remains. In accordance with previous reports, this Payoff-SPN effect seems to have posterior sources, including again parietal areas and the insular cortex.

Therefore, our opening hypothesis that the amplitude of the SPN preceding uncertain upcoming events would be larger than the one preceding predictable events has been supported. The areas responsible for increased activity associated to uncertainty (DLPFC and ACC) are known to be involved in the control and monitoring of learned behavior. Note, however, that control takes place *before the response* (for example, when an individual has to consider the potential costs and benefits of response choices [35]); and monitoring takes place *after the feedback*, that is, when a choice is rewarded/penalized, or a prediction confirmed/disconfirmed [36]–[37]. Non-contaminated psychological uncertainty arises *after the prediction*, but *before the outcome*, that is, when the result of the prediction is at stake.

The posterior parietal areas we have observed to be directly correlated with predictability have also been frequently related to selective attention [38], and memory encoding and recollection [39]. So, posterior parietal areas seem to be involved in codifying the associative evocation or facilitation of the features of the upcoming event. Our data thus suggest that prefrontal (DLPFC and ACC) and more posterior (insular and parietal) cortices could play different roles in a network engaged in expectancy/uncertainty computation. The fact that posterior areas correlate with outcome predictability shows their potential involvement in the associative re-enactment of the perceptual and hedonic properties of the outcome. When these areas fail to generate expectancy, that is, when the outcome has been learnt to be unpredictable, the prefrontal areas are probably responsible for accruing and reacting to uncertainty. This model is compatible with the SEM models depicted in Figure 5. In the best-fitting model for the MaxU condition there appears to be a single active fronto-posterior connection (from the DLPFC to the parietal), whereas in the best-fitting model of the MidU condition, two of the four possible fronto-posterior connections appear to be active. Although the idea is still rather tentative, these connections could implement successful attempts to associatively activate the emotional and perceptual features of the upcoming outcome.

Please note, however, that coactivations – as depicted by our SEM models – do not strictly correspond to anatomical projections among areas. First, the fact that two areas are anatomically connected does not imply that there must be an active connection between the two within the interval of interest (which is only a fragment of the whole period). Our SEM models represent coactivations among areas, and have been induced from the window of interest only. And second, it is important to take into account that both the ACC and the insula are complex structures with several subdivisions. The dorsal part of the ACC (the part activated in our study) is interconnected with the DLPFC, the parietal cortices, and the SMA [40]. The posterior part of the insula, on the other hand, is connected with parietal and temporal cortices, but not that strongly with the ACC [41].

### Integration with Previous Evidence

First, the uncertainty hypothesis is not incompatible with previous studies on the involvement of the prefrontal cortex in learning cue-reward and response-reward associations [35], [42]–[44]. On the one hand, unexpected outcomes are known to generate prefrontal activation that quantitatively mirrors prediction error, and this prediction error is necessary to update the associative link between the cue and the outcome [45]–[47]. However, in the basic associative framework, non-correlated events are assumed to generate no new learning. In contrast with this idea, a number of studies show that non-correlation generates the actual belief that the two events are uncorrelated (learned irrelevance; [48] see also [49]–[50]). In our experiment, non-contingency generated a belief of causal inefficacy, and, simultaneously, an incremented pre-outcome SPN effect. Although the precise role of this uncertainty-related SPN remains speculative, informal reports by our participants in non-contingency conditions reveal attempts to find a way to predict the outcome, alternative to mere conditioned expectancy. Our intuition is that uncertainty motivates such attempts, which generates the prefrontal activity observed in SPN. In that sense, our proposal is that the involvement of prefrontal cortex in uncertainty perception has more to do with learning non-contingency than with learning contingency. This idea in not incompatible with the dominant associative expectancy approach, but certainly complements it.

Second, some studies attribute a direct role in the pre-outcome uncertainty to the midbrain dopaminergic system. For example, Mattox et al. [51] compared the SPN of patients suffering the Parkinson disease (in which the midbrain dopaminergic system is severely damaged) with that of healthy controls, using the weather prediction task. As expected, the SPN in controls was larger for the difficult condition (3 cue cards), than for the simple condition (1 card). More interestingly, the SPN in patients was unrelated to task difficulty. This pattern is compatible, again, with the implication of uncertainty in the SPN, but also points out to the implication of pre-feedback dopamine release in its generation (see [52], for similar results on healthy participants under challenge with the dopamine agonist metilphenidate).

Animal studies corroborate this last idea. Fiorillo et al. [53] registered the activity of midbrain neurons in monkeys, from the onset of the cue to the expected time of the outcome. The function relating expectancy and those neurons’ activity was inverted U-shaped, with the maximal activation for the highest uncertainty and lowest for total certainty (for example when the cue was always followed by the outcome). Interestingly, the activity peak occurred at the time of the expected outcome, which, according to these authors, corresponds to the time of greatest uncertainty (see also [54]).

And third, our results can cast new light on some previous, apparently contrary results. Damen and Brunia [55] used a time estimation task to compare the SPN elicited by knowledge of results and the one elicited by task instructions. The fact that only knowledge of results (KR feedback) elicited a reliable SPN led these authors to conclude that SPN is only caused by upcoming stimuli correlated with the preceding response. Alternatively, it has been proposed that pre-feedback and the pre-instruction SPN are functionally different [56]. However, it seems also plausible to assume that upcoming task instructions do not engage the expectancy brain network. That is, in Damen and Brunia’s control condition people did not learn to predict based on the received instructions, especially because, from the very beginning, it was clearly stated that responses and instructions were unrelated.

In the time estimation task in Kotani et al’s study [9], the stimulus to be predicted varied across conditions in two dimensions: the informational richness of the feedback provided (just correct/incorrect, or more detailed information on the degree of correctness) and the motivational significance of feedback (monetary reward, or just information). The authors observed an increased SPN in the high information/monetary reward condition – when compared with the other three –. The contribution of information richness to SPN is easily explainable in terms of uncertainty. With standard feedback, only two results are possible (correct/incorrect); in the rich feedback condition there are 7 possible results (depending on the degree of correctness), so uncertainty is objectively higher in the rich information condition.

However, the effect of information was significant only in the reward condition (namely, when real money was at stake), which is compatible with the idea that the effect of uncertainty increases when the individual is highly motivated. Nevertheless, motivation and uncertainty are related, but separable dimensions. As noted above, the areas responsible for increased activity associated to uncertainty (DLPFC and ACC) are known to be involved in effortful control. Effort is unnecessary if a prediction is generated easily, via associative evocation, in the low uncertainty condition. And the other way round, if the result is uncertain but motivationally irrelevant, there will be no effort investment either. Both motivation and uncertainty are necessary conditions for the investment of mental effort during the prediction-feedback interval. This connection between motivation and uncertainty justifies the involvement of the dopaminergic system.

However, being motivated to predict an uncertain but hedonically relevant stimulus, and to be able to evocate the hedonic (aversive or appetitive) properties of the upcoming event are essentially different processes (see [10], [57]). Actually, there have been attempts to demonstrate that the SPN is generated by hedonic evocation. For example, in a decision making task, Masaki et al. [8] compared a condition in which people had to learn to select the most profitable choice from an array (the choice condition) against a condition in which they knew in advance rewards were delivered at random (the no-choice condition). As expected, the choice condition elicited a larger SPN than the no-choice one. These results can be interpreted, as authors did, assuming that SPN is an index of expectation for reinforcement. However, it seems also reasonable to assume that only participants in the choice condition were engaged in the computation of the action-reward causal link. So, once more, the level of psychological uncertainty, and not only the level of expected reward, was different in the two conditions.

On this regard, the null results from Ohgami et al [57] using a time estimation task are also at least partially compatible with our interpretation. The main manipulation in that study comprised the hedonic consequences of feedback (only reward, only punishment, combined, or neutral). There was no manipulation of uncertainty in this case, but, strictly speaking, there was no effect of the motivational significance manipulation either (but a laterality x feedback type interaction, by virtue of which interhemispheric differences vanished in the reward condition). As noted earlier, if we take that interaction as evidence of the contribution of hedonic anticipation to SPN, it would not be incompatible with our results, but complementary. Still, the fact that the effect was less intense and more elusive in this case that in other experiments seems to imply that the potential effect of uncertainty (when it is manipulated) on the SPN is larger than the mere effect of hedonic evocation.

Summarizing previous evidence, in most SPN studies carried out to date, there has been a chance for uncertainty to influence the key contrasts. As noted above, for uncertainty to have psychological and physiological entity, the network responsible for expectancy computation must engage in predicting the outcome, and then fail to do so. However, it is important to acknowledge that the prefrontal areas showing more activity in our high uncertainty condition, and the ones involved in representing expected rewards are not overlapping, so the previously cited results are complementary, rather than contradictory, with ours.

Our results are not incompatible with the involvement of prefrontal cortex in computing prediction error, but, at the same time, points out to the insufficiency of the expectancy generation/violation mechanism assumed to be responsible for learning in all associative models. As noted above, both animal and human studies had already found that uncertainty is quantitatively related with pre-outcome dopaminergic activity. Our results demonstrate that prefrontal structures are also part of this brain mechanism of uncertainty computation.

### Conclusion

The main result of the present study is the demonstration that psychological uncertainty crucially contributes to SPN, and the potential involvement of DLPFC and ACC in the generation of such psychological and physiological activity. Thus, the SPN can be considered a manifestation of part of a broad mechanism for adaptive behavior. At a cortical level, the network responsible for expectancy/uncertainty generation would include the anterior cingulate and dorsolateral prefrontal cortices, posterior parietal areas, and the insula. Part of this network is probably responsible for generating expectancy, that is, the anticipated representation of the hedonic and perceptual features of the upcoming event. However, there is another part, the most anterior one, coming into action when the learner has been unable to predict the outcome in the past, or, what amounts to be the same, when the upcoming event is perceived as uncertain. Consequently, the SPN arises as a tool to study the interaction of uncertainty with other processes, and, more specifically, the role of uncertainty in normal and pathological decision making, a possibility recently proposed in the fields of behavioral economics and neuroeconomics [58]–[59].
